# A Substitute for Quinine

**Published:** 1890-07-12

**Authors:** 


### A SUBSTITUTE FOR QUININE.

Mons. Valude professes to have found a substitute for quinine in *Panbatano*, a tree indigenous to Mexico, of the family of Leguminosæ. The preparation employed by Mons. Valude was made by macerating 70 parts by weight of the crushed bark in 1,000 parts of water, which was boiled down to one-half the volume. Of this, one pint was taken in divided doses every twenty-four hours. Dr. Valude having administered the preparation in various cases of intermittent fever considers that the action is that of quinine. It is stated that the panbatano contains neither alkaloid nor glucoside. The number of cases, however, in which the remedy was used is not sufficient to demonstrate its efficacy, especially when it is recollected that ague is a malady tending to spontaneous decline. It is this characteristic of ague which has caused so many things to be credited with anti-periodic powers. Ague has declined after the use of such things, *ergo*, all are anti-periodics! Even quinine has attained much credit to which it is not entitled, from the decline of ague *after* its "exhibition," and perhaps not *because* of it. Nearly every drug in the pharmacopœia has at one time or other been vaunted as an anti-periodic. Arsenic, opium, iodide of potassium, the salycilates, fuming nitric acid, nitrate of potash, and many other things, have all had advocates. Substances also, not in the pharmacopœia, have been credited with anti-periodic powers; for instance, cob-webs and black coffee. We therefore look with some critical distrust on any agent which may be recommended as a substitute for quinine.

